# Spatial Characterization of Electrogram Morphology from Transmural Recordings in the Intact Normal Heart

**DOI:** 10.1371/journal.pone.0110399

**Published:** 2014-10-31

**Authors:** Jim Pouliopoulos, William Chik, Karen Byth, Elizabeth Wallace, Pramesh Kovoor, Aravinda Thiagalingam

**Affiliations:** 1 Department of Cardiology, Westmead Hospital, Sydney, Australia; 2 The University of Sydney, Sydney, Australia; University of Minnesota, United States of America

## Abstract

**Purpose:**

Unipolar (UE) and bipolar electrograms (BE) are utilized to identify arrhythmogenic substrate. We quantified the effect of increasing distance from the source of propagation on local electrogram amplitude; and determined if transmural electrophysiological gradients exist with respect to propagation and stimulation depth.

**Methods:**

Mapping was performed on 5 sheep. Deployment of >50 quadripolar transmural needles in the LV were located in Cartesian space using Ensite. Contact electrograms from all needles were recorded during multisite bipolar pacing from epicardial then endocardial electrodes. Analysis was performed to determine stimulus distance to local activation time, peak negative amplitude (V_-P_), and peak-peak amplitude (V_P-P_) for (1) unfiltered UE, and (2) unfiltered and 30 Hz high-pass filtered BEs. Each sheep was analysed using repeated ANOVA.

**Results:**

Increasing distance from the pacing sites led to significant (*p*<0.01) attenuation of UEs (V_-P_ = 7.0±0.5%; V_P-P_ = 5.4±0.3% per cm). Attenuation of BE with distance was insignificant (V_p-p_ unfiltered  = 2.2±0.5%; filtered  = 1.7±1.4% per cm). Independent of pacing depth, significant (*p*<0.01) transmural electrophysiological gradients were observed, with highest amplitude occurring at epicardial layers for UE and endocardial layers for BE. Furthermore, during pacing, propagation was earlier at the epicardium than endocardial layer by 1.6±2.0 ms (UE) and 1.4±2.8 ms (BE) (all *p*>0.01) during endocardial stimulation, and 2.3±2.4 ms (UE) and 1.8±3.7 ms (BE) during epicardal stimulation (all *p*<0.01).

**Conclusions:**

Electrogram amplitude is inversely proportional to propagation distance for unipolar modalities only, which affected V_-P_>V_P-P_. Conduction propagates preferentially via the epicardium during stimulation and is believed to contribute to a transmural amplitude gradient.

## Introduction

Interpretation of electrograms during electrophysiological mapping of arrhythmias provides critical insight relating to the nature of their underlying substrates. Electrogram amplitude recorded utilizing either bipolar or unipolar modalities of mapping traditionally infer information regarding the magnitude of surviving myocardium in proximity to the recording electrodes. Substrate mapping is often performed during pacing from a variety of strategic locations when stable sinus rhythm is not possible [1].

Analysis of the three-dimensional spread of electrical activation during stimulation and the effects from anisotropy on unipolar electrograms have been previously described [2]–[5]. It is well documented that the greatest negative potential occurs closest to the site of pacing in normal myocardium [2]. During the initial stages of evoked activation and at short distances from the stimulus origin, the potential gradient is affected by local tissue anisotropy [3]–[5]. With greater distance from the stimulus site, we hypothesize that the electrogram amplitude gradient becomes increasingly homogenous and less influenced by the underlying anisotropic structure of myocardium in normal hearts [3], [5].

The *In Vivo* dynamics of long-distance myocardial propagation on unipolar and bipolar electrogram amplitude in an intact heart during multisite epicardial and endocardial pacing with simultaneous transmural multisite recordings has not been previously quantified in detail.

This study aimed to (i) quantify the effect of distant epicardial and endocardial stimulation on local unipolar and bipolar electrogram amplitude and (ii) determine whether electrogram amplitude gradients exist between transmural layers of myocardium with respect to propagation and depth of stimulation.

## Methods

### Animals Used for the Study

Experiments were performed on 5 male castrated sheep 45±7 kg. The study was approved by the Animal Research Ethics Committee of Westmead Hospital. The study protocol conformed to the guidelines set for animal research by the National Health and Medical Research Council, Australia.

### Procedural Setup

Following premedication with Xylazine (40 mg) and Atropine (500 µg) intramuscularly, anaesthesia was induced using intravenous pentothal (450 mg). An endotracheal tube was inserted and mechanical ventilation commenced using a Harvard Dual Phase Control Respirator Pump. Anaesthesia was maintained with isoflurane (1–3%) in 100% oxygen.

**Figure 1:**
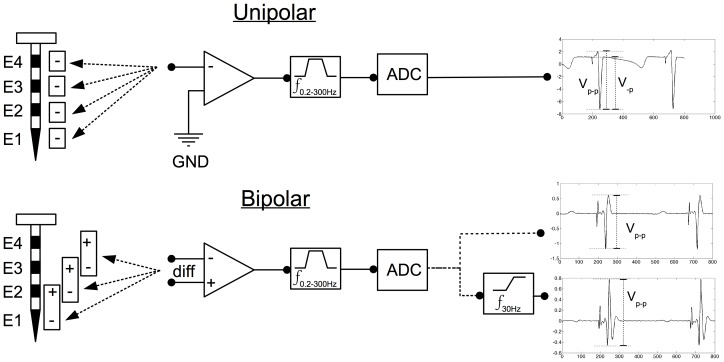
Illustration of electrocardiographic recording method, signal processing, and measurement of electrograms. **E1–E4**  =  endocardial – epicardial plunge needle electrodes. +/−  =  electrode terminals. **Diff**  =  differential of +/− terminals. **Triangle**  =  Analogue Amplifier. **GND**  =  Ground Terminal attached to rib-retractors. **ADC** =  Analogue to Digital Converter (1 kHz sampling). **Step Functions** (**f**): Band-Pass filtering in analogue domain (0.2–300 Hz) and High-Pass filtering in the digital domain (not performed or 30 Hz). **V_-P_**  =  Maximum negative deflection amplitude of QRS complex. **V_P-P_**  =  Maximum peak positive to peak negative deflection amplitude of QRS complex.

A left thoracotomy was performed through the 4th intercostal space. The heart was exposed, and a grid of 50–60 multi-electrode mapping needles (0.8 mm diameter, four 1.5 mm length electrodes separated by 1.5 mm length Teflon spacers) was positioned through the anterior and lateral left ventricle (see figure 1). The needle at the top left corner of the grid was positioned immediately below the atrioventricular groove. The first row of needles was then placed along the atrioventricular groove with an inter-needle spacing of 1 cm. Subsequent rows were added at 1 cm intervals below and parallel to the first row.

Needle electrodes were ordered as distal (Endocardial, E1) to proximal (Epicardial, E4). The electrodes E2 and E3 recorded electrograms from the mid myocardium, whilst E1 recorded electrograms from the endocardium when in physical contact. An electrode attached to the rib retractors was used as the unipolar reference.

Following insertion of all needles, heparin was administered I.V (5000 IU), followed by hourly maintenance doses (1000 IU/hour) for the duration of the case. The epicardial surface of the heart was routinely moistened with 0.9% w/v NaCl spray throughout the procedure.

An Ensite multi-electrode array (MEA) was introduced via the retrograde aortic approach into the left ventricle and positioned at the apex and inflated to 90% of maximum profile. The Ensite system determined the location of each distal needle electrode in Cartesian space by passing a 5.6 kHz signal via that electrode and measuring the subsequent signals from each of the 64 electrodes within the MEA (Enguide locator system, Endocardial Solutions, St. Paul, MN, USA). Reconstructed non-contact electrograms were not analysed for this study but were incorporated into figure 1 to illustrate the pattern of activation across intramural contact electrodes during stimulation from the apex. Limitations of unipolar noncontact electrograms in ventricular electroanatomical mapping procedures have been reported previously [6], [7].

### Electrophysiology Study

After a 30 minute settling period, baseline transmural unipolar electrograms from all of the needles were recorded simultaneously using a Prucka Cardiomap 256 channel mapping system (Prucka Engineering, Houston, TX, USA) [8]. Unipolar electrograms with high signal to noise ratios were recorded using high gain amplification at a sampling frequency of 1 kHz with a filtering bandwidth of 0.2 to 300 Hz.

Pacing at twice the diastolic threshold and cycle length of 400 ms, from each needle site was performed using a Micropace EPS320 cardiac stimulator (Micropace Corporation, Canterbury, NSW 2193, Australia). Bipolar pacing was first performed from the endocardial electrodes (E1–E2), and then repeated for epicardial electrodes (E3–E4). The distal electrodes were configured as the cathode and proximal as the anode during stimulation. During pacing, unipolar electrograms were recorded simultaneously from all plunge needles.

Immediately after completion of the study, the animals were euthanized with an overdose of pentobarbital sodium (300 mg/mL). After 20 minutes, following confirmation of mortality, transmural sections were taken from each needle site. Specimens were fixed in formalin 10% and prepared for optical microscopy. For histological analysis hematoxylin and eosin staining was performed.

Unipolar electrograms recorded during multisite pacing and their corresponding needle coordinates were exported into an in-house customised software utilising the Matlab programming language (Matlab V7, Mathworks) for offline analysis.

### Definitions for Electrograms

Illustrations of the needle electrode configurations for bipolar and unipolar recordings, and signal processing methods are outlined in figure 1.

Analysis of local activation time (AT), and electrogram amplitude was performed on minimally filtered (0.2–300 Hz bandpass) unipolar and bipolar electrograms spanning paced beat sequences that did not have atrial activation within ±100 ms of the QRS signal. Additional analysis was performed on bipolar electrograms, which were high-pass filtered at 30 Hz.

Bipolar electrograms at different intramural layers were constructed by subtracting proximal from distal electrode unipolar electrogram recordings (E12  =  endocardial; E23  =  mid myocardial; E34  =  epicardial).

For unipolar electrograms, activation time (AT) was defined as the time interval from the stimulus spike to the QRS dV/dt_min_. To minimise the requirement of filtering for baseline wander of the electrograms, the iso-voltage was defined as the mean voltage spanning 10 ms prior to the stimulus spike or p-wave. If the voltage at any given time within the 10 ms window extended beyond 2 S.D of the mean, the window was rejected, and an alternative window spanning a different region was chosen. Peak negative (V_-P_), and peak to peak (V_P-P_) voltages were calculated as QRS amplitudes. For V_-P_, the iso-voltage was standardised as 0 voltage.

For bipolar electrograms, activation time was defined as the time interval from the stimulus spike to absolute maximum QRS amplitude [9]. Bipolar V_P-P_ was calculated as the peak-peak amplitude of the signal spanning the QRS segment.

The distance between the stimulus site, and the local activation site was calculated using the Cartesian distance formula.
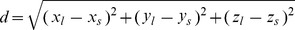



Where *d* =  distance in mm; *l* =  local measurement site; *s* =  location of stimulus site; and (*x,y,z*)  =  Cartesian coordinates in three dimensions.

### Statistical Analysis

The data recorded from each sheep consisted of recordings from 60 needles with 4 unipolar electrodes (total of 1200 unipolar electrograms) and 3 unipolar electrode pairs (total of 900 bipolar electrograms) at differing depth from the epicardium and distance from the source of pacing.

Electrogram criteria were analysed after transforming (log_10_) the raw data to approximate normality. Subsequently, each sheep was analysed separately using within-subjects, repeated analysis of variance using Huynh-Feldt correction. In the model, depth of the electrodes and pacing location (E12 and E34) were considered as factors, and distance as a covariate. The within-subject variables included V_-P_, and V_P-P_. Two-tailed comparison was used to test for similarities in the variables/distance dynamics measured between sheep at individual myocardial depths.

For analysis of activation time, differences in activation time between epicardial and endocardial layers (unipolar E4–E1; bipolar E34–E12) from each sheep were aggregated based on stratified distances from the stimulus site (increments of 20 mm), and depth of bipolar stimulation (E12, and E34). One-sample t-tests were then performed to assess whether differences in activation times between each of the myocardial layers assessed, departed significantly from 0 ms.

For all analyses, differences were considered significantly different at an *α* level of 0.05 or less. All statistical analysis was performed using the Statistical Package for the Social Sciences (SPSS) for Windows (Release 12, SPSS, Inc., Chicago, IL, USA).

## Results

**Figure 2:**
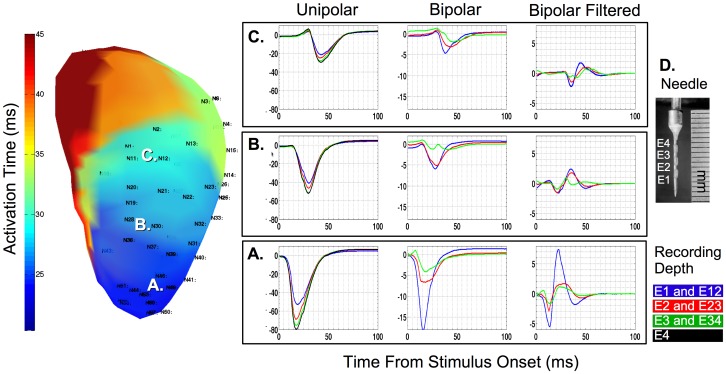
Left: Three dimensional electroanatomic map of the left ventricle (anterior projection) displaying activation time derived from non-contact electrograms during pacing from a needle (D) located at the left ventricular apex. The figure shows earliest activation (dark blue) at the apex with gradual spreading towards the base (red). Numbers on electroanatomic map (fineprint) represent randomised locations of multipole needles (shown rightmost), which were deployed via the epicardium. **Right: Panels A, B and C demonstrating transmural unipolar and bipolar contact electrograms recorded from multipole needles corresponding to sites A, B and C on the electroanatomic map.** Y-axis is represented in mV. Electrodes; E1–E4  =  endocardial-epicardial unipolar recording depth; E12–E34  =  endocardial-epicardial bipolar recording depth.

Tissue depth related changes in unipolar electrogram amplitude and morphology depended on the distance of the recording site from the site of stimulation [Figure 2]. Corresponding bipolar electrograms from the same locations and depths indicated that small differences in unipolar morphology between adjacent electrodes contribute to marked increases in electrogram amplitude of minimally filtered bipolar signals. This increase in electrogram amplitude was preserved after high pass filtering. Conversely spatial similarities in unipolar electrogram morphology between adjacent electrode pairs resulted in a low amplitude bipolar signal.

As expected, unipolar electrograms recorded in proximity to pacing sites exhibited qS morphology, whereas electrograms from distant sites exhibited morphology with R-wave characteristics. Inferences to distance were not visually distinguishable from bipolar electrogram amplitude or morphology [Figure 2].

The effect of propagation distance and transmural recording depth on electrogram amplitude is illustrated in Figure 2 and statistics are summarised in Table 1 for unipolar and Table 2 for bipolar modalities. Respectively, table summaries of the interaction effect depth × distance indicate whether propagation distance had an effected on electrogram amplitude at each intramural layer. The interaction effect depth × pacing depth summarises the effect of pacing depth on electrogram amplitude at each intramural layer.

**Table 1:** Within subject analysis examining the effect of propagation distance (covariate), transmural recording depth (treatment), and pacing depth (treatment) on minimally filtered unipolar electrogram parameters measured.

Parameter	Sheep	Amplitude Attenuation (%) per cm	ANOVA P-Value		
			Depth	Depth × Distance	Depth × Pacing Depth
Unipolar V_-P_	1	6.6	<0.01	<0.01	0.89
	2	7.9	<0.01	0.01	0.92
	3	6.9	<0.01	<0.01	0.96
	4	6.1	<0.01	0.02	0.75
	5	6.7	<0.01	<0.01	0.49
	Mean	7.0±0.5			
Unipolar V_P-P_	1	5.5	<0.01	0.84	0.96
	2	5.9	<0.01	<0.01	0.81
	3	5.2	<0.01	<0.01	0.80
	4	5.1	<0.01	0.02	0.75
	5	5.1	<0.01	<0.01	0.59
	Mean	5.4±0.3			

Probabilities for the interaction term Depth*Distance represents whether there was a significant effect of propagation distance on electrogram amplitude recorded at each intra-myocardial depth level. Probabilities for the interaction term Depth*Pacing Depth indicate whether pacing depth had a significant effect on electrogram amplitude recorded at different intra-myocardial depth levels. Mean ± SD where indicated.

**Table 2:** Within subject analysis of the effect of propagation distance (covariate), transmural recording depth (treatment), and pacing depth (treatment) on unfiltered and 30 Hz high-pass filtered bipolar electrogram parameters measured.

Parameter	Sheep	Amplitude Attenuation (%) per cm	ANOVA P-Value		
			Depth	Depth × Distance	Depth × Pacing Depth
Bipolar V_P-P_ Unfiltered	1	2.0	0.02	0.15	0.99
	2	1.8	0.07	0.66	0.61
	3	2.8	<0.01	0.01	0.71
	4	2.4	0.68	0.09	0.87
	5	1.7	<0.01	0.09	0.91
	Mean	2.2±0.5			
Bipolar V_P-P_ Filtered	1	1.2	0.24	0.24	0.97
	2	1.5	<0.01	0.34	0.71
	3	2.3	<0.01	<0.01	0.69
	4	1.8	0.12	0.28	0.84
	5	1.3	<0.01	0.06	0.88
	Mean	1.7±0.4			

See Table 1 for interpretation of ANOVA probabilities.

Left ventricular wall thickness at needles sites spanning the apex, equator, and base was 15.6±4.7 mm, 15.8±3.3 mm, and 16.9±4.55 mm respectively. Pacing threshold was <1 mA for all sights. The average distance of needle electrodes to the ensite MEA at apical, equatorial and basal sites was 33.1±4.8 mm, 26.2±5.6 mm, and 35.9±8.9 mm respectively. All electrodes were within distances of less than 50 mm from the ensite MEA.

### Spatial Attenuation of Electrogram Properties during Propagation

**Figure 3:**
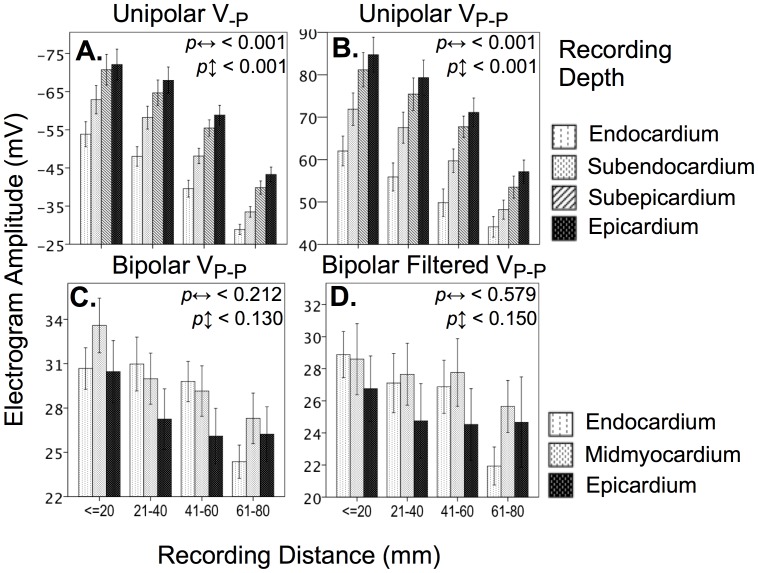
Plots showing effect of recording distance from stimulation source and recording depth on contact electrograms measured during multisite pacing of left ventricular myocardium. There was a significant inverse relationship between recording distance from stimulus location and unipolar electrogram amplitude that was not significant for bipolar electrograms. Values represent between-sheep mean ± S.E, where; *p* ^↔^ is the probability of effects relative to propagation distance; and *p* ^↕^ is probability of transmural effects. A. Unipolar V_-P_; B. Unipolar V_P-P_; C. Bipolar V_P-P_; and D. Filtered bipolar V_P-P_. See figure 1 for electrode locations.

Increasing distances from the pacing sites resulted in significant attenuation of both V_-p_ and V_P-P_ for unipolar electrograms recorded during multisite pacing in 5/5 and 4/5 sheep respectively. (Figure 3 and Table 1). Whilst this effect was observed to a smaller degree on filtered and unfiltered bipolar electrograms it did not reach statistical significance. Attenuation of amplitude with increased distance was far more dramatic and consistent for unipolar electrograms (Figures 2 and, 3; Tables 1 and 2).

**Figure 4:**
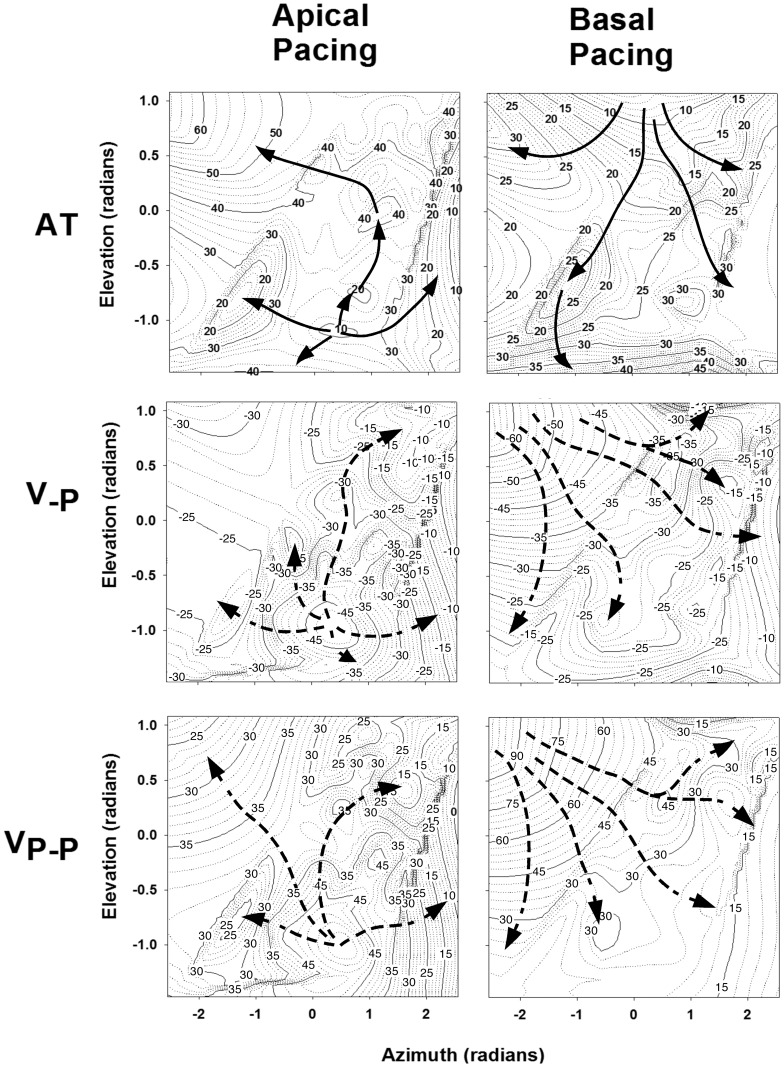
Spatial representation of sub-endocardial electrogram parameters during sub-endocardial pacing from the apex (left panels) and base of the left ventricle (right panels). Two dimensional space is based on spherical coordinates derived from needle locations, representing elevation (−1 =  apex to +1 =  base), and azimuth (−2 =  mid lateral to +2 =  anterior). Numbered contours represent parameters derived from unipolar contact electrograms represented in milliseconds (ms) for activation time (AT) and millivolts (mV) for V_-P_ and V_P-P_ corresponding to panels from top to bottom respectively. Solid arrows represent preferential path of activation. Dotted arrows represent the approximate electrophysiological gradient of V_-P_ and V_P-P_ away from the stimulus site.

Propagation distance affected unipolar V_-P_ to a greater extent than V_P-P_, amounting to a relative amplitude reduction of 56% and 43% respectively, at distances of 8 cm from the stimulus source. In contrast, unfiltered and filtered bipolar electrograms exhibited a relatively minor 18% and 14% reduction in amplitudes of V_-P_ and V_P-P_ respectively, recorded at the same distances. Representation of unipolar V_-P_, and V_P-P_ during pacing from the cardiac apex and base is illustrated in figure 4. Corresponding to the sequence of activation during stimulation from the apex, attenuation of these parameters is observed towards the base, whereas the converse occurred during basal pacing.

### Measurement of Transmural Electrogram Amplitude Gradients

A significant epicardial to endocardial gradient of diminishing amplitude was observed in all sheep for unipolar electrograms using V_-P_ and V_P-P_ measurements (depth; all *p*<0.01). Conversely, a transmural amplitude gradient was observed consistently in only 3/5 sheep for bipolar electrograms. However, the transmural direction of the unipolar gradient was opposite in orientation of the bipolar gradient (figures 2, and 3) which were unaffected by pacing depth in all sheep (Table 1 and 2; recording depth × pacing depth; all *p = *NS).

**Figure 5:**
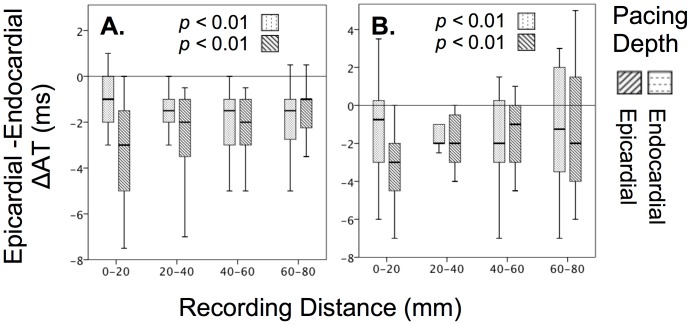
Transmural comparison of activation time between the epicardial and endocardial layer (shown as difference ΔAT) at various distances from the stimulus site during endocardial and epicardial pacing. Comparisons shown are: A. Unipolar and B. Bipolar minimally filtered electrograms.

The transmural electrophysiological gradient observed for unipolar electrograms was consistent with preferential conduction via the epicardium during left ventricular stimulation (Figure 5). During endocardial pacing, the epicardium activated earlier than the endocardium by 1.6±2.0 ms (*p*<0.01) for unipolar and 1.4±3.1 ms (*p*<0.01) for bipolar modalities. Earlier epicardial activation was also observed during epicardial pacing, with significant transmural activation time differences of 2.3±2.4 ms (*p*<0.01) and 1.8±3.7 ms (*p*<0.01) for both unipolar and bipolar modalities respectively. Respectively, during pacing, conduction velocity was greater during transmural propagation, than during propagation parallel to the ventricular wall by a ratio of 1 to 0.7.

**Figure 6:**
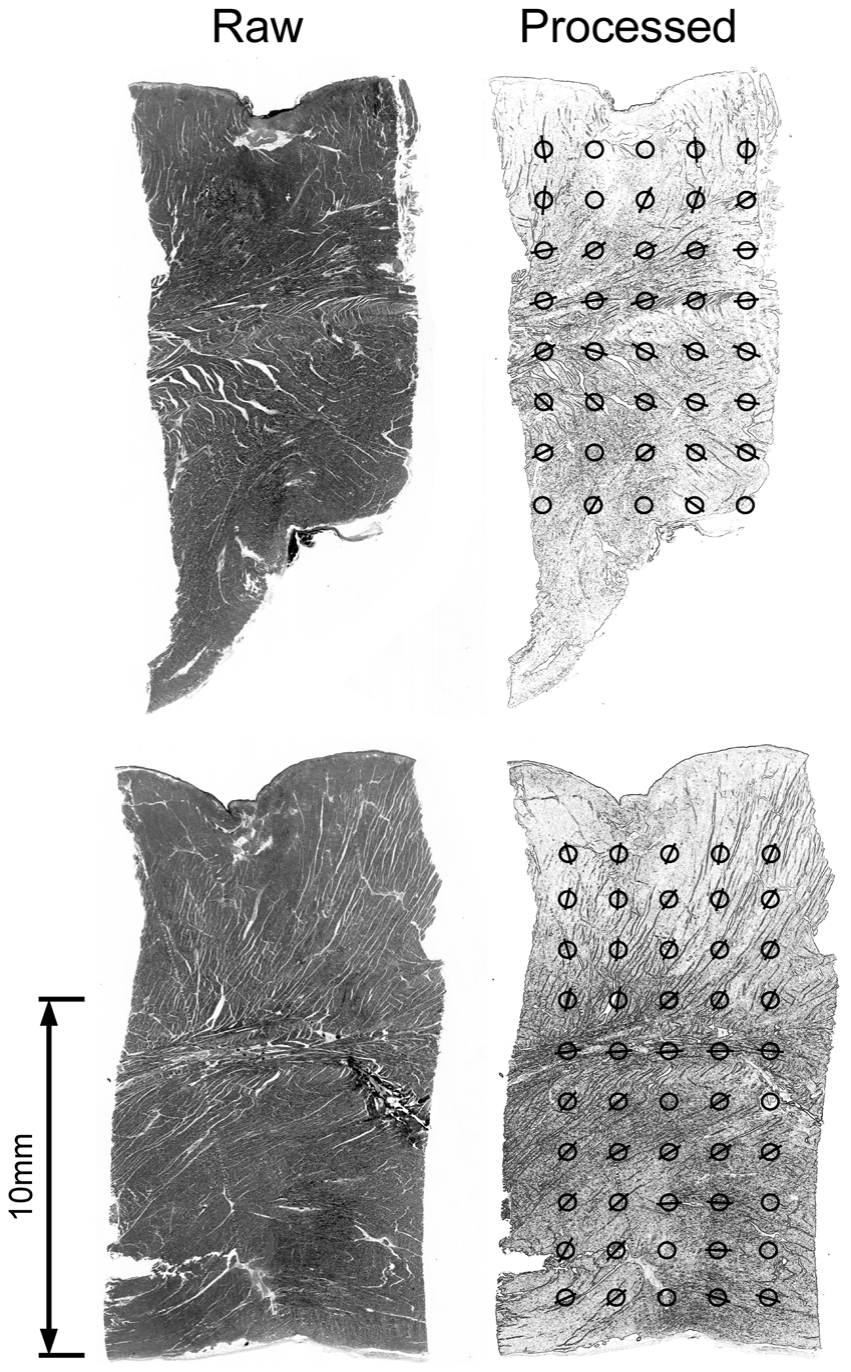
Two representative stained histological sections using haematoxylin-eosin of normal myocardium from different sites in the Ovine left ventricle. Left: unprocessed image. Right: convolved images identifying transmural anisotropy vectors in two dimensions from multiple sites indicated (circles) within each section. Circles arranged from epicardium (top) to endocardium (bottom). Anisotropy vectors orientated vertically at epicardial layer with distinct transitioning to horizontal orientation at the mid myocardial layer followed by diagonal orientation at the endocardial layer.

Upon histological examination of the left ventricular wall, three distinct orientations of the myofibre architecture were observed (Figure 6). Tissue processing resulted in an approximate 10% reduction in volume. The cellular orientations were readily observable because the reduction in volume post processing resulted in separation of myocytes at cleavage sites, thus revealing two orthogonal bands of myofibres. At the epicardial layers, the myofibres were distinctly organised in a normal (epicardial-endocardial) vector to the plane of the myocardial layers. In contrary, fibre orientations were distinctly parallel at the mid myocardium while anisotropy at the endocardium level displayed greater inhomogeneity.

## Discussion

This *in vivo* study, which examined the transmural effects from myocardial pacing on unipolar and bipolar electrograms quantitatively in a normal ovine model, is the first of its kind to the best of our knowledge. Our results demonstrated that unipolar electrogram amplitude, in contrast to bipolar electrogram, decayed significantly as propagation distance from stimulus site increased. These findings are of immediate clinical and scientific relevance. The clinical significance of these observations impacts on how unipolar electrograms may be interpreted when utilized for clinical substrate mapping studies. We found that propagation distance affected unipolar V_-P_ more than V_P-P_ suggesting that V_P-P_ should be considered as a preference over V_-P_ during unipolar substrate mapping studies.

Secondly, we have demonstrated that preferential conduction occurs via the epicardium during pacing in sheep hearts. This effect coincided with a transmural electrophysiological gradient, resulting in the greatest unipolar electrogram amplitudes being observed at the epicardial layers whereas the greatest bipolar electrogram amplitudes were ascertained at the mid-myocardial and endocardial layers. Histological evidence suggests that the preference of the epicardium to activate earlier during ventricular paced rhythm may be dependent on epicardial fibre orientation and transmural segmentation by parallel orientation of mid myocardial fibres. The change in fibre orientation at the mid myocardial layer and a preference in conduction along the epicardium may contribute to increased transmural electrogram heterogeneity. From these findings, we hypothesise that preferential conduction of the epicardium may also occur during ventricular tachycardia at sites distant from the focal source of initiation.

Fundamentally, the unipolar electrogram is generated by active and passive properties of the myocardium during activation and recovery. Active currents are responsible for maintenance of a resting membrane potential and cellular depolarisation leading to an action potential. This in turn is dependent on an electrochemical gradient created by cell-to-cell transmission of electrically charged ions through gap junctions. Passive currents attributing to the electric field within and surrounding the heart can be modelled as a dipole where the site undergoing depolarisation is a current source. The current flow is directed outwardly towards neighbouring myocardial cells in the resting state. Hence, the distribution of extracellular potential at or near the site of depolarising myocardium gives rise to a negative deflection on the unipolar electrogram. On the contrary, unipolar electrogram potential becomes positive when depolarisation occurs at distant sites or sites depolarising the latest. Far field electrical activity in the heart can further increase the complexity of unipolar electrogram interpretation.

On the other hand, bipolar electrograms are representative of the spatial summation of adjacent signals and hence are useful for identifying areas of spatial heterogeneity of electrophysiological recordings. Bipolar electrograms are commonly employed during clinical electrophysiological mapping studies as the far field effects are cancelled out from local recordings. The precisions of bipolar electrograms however, are fundamentally dependent on the orientation of the electrode pairs relative to the vector of the propagation wavefront. In this study, all electrode pairs were orientated perpendicular to the ventricular wall in order to provide a consistent bipole angle with respect to wavefront propagation along the ventricular wall.

We assessed electrogram amplitude of bipolar high pass filtered electrograms in this study because they are commonly employed in clinical electrophysiology procedures. Unipolar electrograms were minimally filtered to conserve morphology relating to spatiotemporal propagation.

### Effect of Distance from Stimulation Site on Local Electrogram Amplitude

Our observations indicate that negative and peak-peak electrogram amplitudes for unipolar electrograms were significantly affected by propagation distance from the source of stimulation at all intramural layers. However, bipolar electrogram amplitude was not significantly influenced during propagation away from the stimulus sites. This suggests there was no significant overall change in spatial heterogeneity as propagation wavefront proceed away from pacing site.

While our results were consistent with prior qualitative *In Vivo* and computer simulation based studies regarding extracellular potential distributions spatially in relation to stimulation proximity, this is the first study to quantitatively assess these effects over greater distances and systematically using combined observations of the different recording modalities from *In Vivo* transmural plunge needle measurements [4], [10], [11]. Our results indicate that the local unipolar electrogram amplitude is attenuated over much greater distances in the heart than what has been demonstrated in previous studies. Furthermore, this relationship was consistently evident in the majority of hearts studied.

Essentially, a crucial distinction exists between local unipolar peak-positive and peak-negative voltage relative to the distance of propagation, such that the effect of distance was less for peak-peak compared to peak-negative voltage. The reason for this is beyond the scope of this study, yet represents an important factor to consider during clinical voltage mapping procedures using unipolar electrogram recordings. Possible explanations relating to attenuation of unipolar V_-P_ include conductivity changes associated with tissue anisotropy and effects relating to bifurcation of conduction [3], [5], [12]. These effects have not been previously investigated in large volume conductors beyond the millimetre scale. Observations from Kucera *et al*., demonstrated that branching along linear strands of interconnecting myocytes resulted in conduction slowing along the strand causing subsequent reduction in action potential upstroke amplitude [12]. Such changes to the action potential are likely to influence electrogram morphology. The phenomenon mentioned by Kucera *et al.*, and others is known to occur as a result of electrotonic effects by passive transmission of current from neighbouring myocytes via low resistance pathways [12], [13]. At those small scale, electrotonic effects decay exponentially during propagation such that the ultimate effect on membrane conductance becomes negligible at greater distances away from the initial stimulus [14].

### Transmural Electrogram Amplitude

Our results illustrate the presence of a distinct electrophysiological gradient of diminishing unipolar electrogram amplitude associated with increasing depth towards the endocardium. A similar transmural gradient exists for bipolar electrograms, but its orientation was opposite to unipolar signals. This might be related to the greater variations in unipolar electrogram amplitude observed between the mid-myocardial to endocardial electrodes than between the subepicardial to epicardial electrodes

In *Canine* hearts Frazier *et al.*, observed that during sub-epicardial or sub-endocardial pacing, the unipolar amplitudes away from the local stimulation zone were larger at the sub-epicardium than sub-endocardium, while sub-endocardial potentials local to the site of pacing were larger in amplitude [15]. Compared to our methods, their study was limited to mapping only a small volume of myocardium (20×35×5 mm) within the lateral right ventricle, thereby potentially omitting critical transmural gradients that might have existed beyond the initial site of stimulation.

Computer modelling, limited to small myocardial slab volumes, showed that endocardial potentials could be enhanced by the absence of an intracavity blood boundary [2], [16], [17]. Increasing electrogram amplitude at the endocardium has been demonstrated in *Canine* hearts when conductivity of the surrounding medium is reduced [18]. This was later observed in patients undergoing antiarrhythmic surgical procedures, and from experiments of langendorf perfused *Porcine*, as well as *Canine* left ventricular preparations, where the absence or presence of intracavity blood affected both R wave and peak-peak unipolar amplitude [19]. However, little is known about the effect on conduction in the absence of intracavity blood. Electrogram amplitude can be altered by changes to the action potential, which could result from an imbalance of autonomic innervation manifested by a spectrum of variables including changes in heart rate, blood pressure or mechanical volume load [20], [21]. In the previously mentioned study by Potse *et al.*, distinct differences in endocardial activation time were observed between filled and empty ventricular chambers [19]. Hence, the transmural electrophysiological gradient observed is unlikely to be a phenomenon limited to the ovine model. In support of this hypothesis, Plonsey *et al.*, demonstrated an increase in extracellular potential with increasing depth from the endocardium [22]. Their study employed bidomain model simulations where the endocardium was bounded by blood, and transmural depth was extended semi-infinitely. This indicates that the transmural electrophysiological gradient measured in that study occurred in the absence of an open-to-air epicardial interface.

Therefore, it is highly likely that the spatial arrangement of myolaminae or intramural laminar discontinuities give rise to transmural dispersion of electrogram morphology and amplitude. Hook *et al.*, demonstrated the importance of myolaminae anisotropy and transmural variation in conductivity on influencing electrical field within the heart in a *In Vivo* porcine study [23]. This was achieved through utilising conductivity measurements sampled from a small area of the lateral ventricle which were correlated with MRI and histological tissue ultrastructure to mathematically simulate the cardiac electrical field.

We confirm and extend this knowledge by reporting on transmural unipolar and bipolar electrogram measurements performed *In Vivo* over the entire left ventricular wall, where recordings were determined with accurate localisation and reproduced consistently by pacing from multiple sites across multiple animals.

### Preferential Conduction

Experimental modelling of 3-Dimensional myocardium from macrostructural analysis has shown that initial propagation from a point focus is significantly influenced by the local laminar tissue structure. [5], [24], [25] Those studies accomplished detailed observations and predictions about propagation within close proximity to the stimulating electrodes but have overlooked aspects of propagation spanning a greater mass of myocardium at greater distances from the stimulation sites. The effect of the virtual electrode on local propagation may be a confounding influence in those studies. In this study, we were able to map in detail, *In Vivo* electrogram characteristics during propagation distances of approximately 80 mm over a region spanning the left ventricle with exclusion of the septum and postero-lateral wall.

Based on the oblique dipole layer model derived from isolated heart measurements, as the propagation wavefront travels further away from the stimulus source, the impact of laminar structure is proportionately reduced [2], [5]. From our study, this effect was evident with a 13% reduction in bipolar electrogram amplitude seen at 60 mm from the stimulation source. This suggests the spread of activation at such distances becomes increasingly homogenous. However, evidence from both unipolar and bipolar recording modalities in this study indicate that the propagation wavefront, always reaches the epicardial layer first despite initial activation of the endocardial or epicardial layers.

The preference for epicardial conduction appears to be specific to invoked stimulation from within ventricular myocardium. Human and canine ventricular activation typically occurs from endocardium to epicardium during sinus rhythm [15], [26], [27]. In contrary, a reversed transmural gradient of activation was demonstrated in porcine hearts using plunge needle mapping [26]. While the transmural activation sequence during multisite pacing has not been evaluated systematically in humans, it was recently identified in canine hearts that abnormal transmural activation can occur throughout the left and right ventricles depending on the site of stimulation [28]. In this study, the transmural pattern of activation in the left ventricle was reproducible within all sheep. Furthermore, this study showed a distinct parallel orientation of mid-wall myofibres, suggestive of mid-myofibre rotation. The location of this band separated the homogeneously anisotropic epicardial layers from the inhomogenously anisotropic endocardial layers. The increased mid-myocardial to endocardial bipolar electrogram amplitude confirmed the increase in electrophysiological heterogeneity at these locations.

The myofibre architecture of the left ventricle observed in the ovine model reported in this study and others is consistent with histological and ultrastructural studies of human hearts [3], [5], [29], [30]. We hypothesize that the orientation of mid-wall and epicardial fibres may play a role in coordinating the transmural pattern of propagation observed in the absence of purkinje activation.

## Conclusions

Unipolar electrogram amplitude decreased proportionally with increasing propagation distance which affected V_-P_>V_P-P_. In contrast, bipolar electrogram amplitude was spatially resistant to changes in propagation distance along the ventricular wall. Preferential epicardial conduction, observed during stimulation, and transmural anisotropic changes, resulted in transmural unipolar and bipolar electrogram amplitude gradients.

### Clinical Relevance

Situations where ventricular pacing is necessary during substrate mapping procedures includes patients that are pacemaker dependent, have frequent ventricular ectopic beats or in atrial fibrillation with varying P-R intervals. Unlike bipolar mapping modalities, the effect of tissue transmurality and distance from the site of earliest activation have implications in unipolar modality mapping of myocardial scar utilising electrogram amplitude. This does not implicate unipolar mapping to be inferior to bipolar mapping. This effect, however, suggests that propagation distance and recording depth effects should be considered for interpretation of unipolar electrograms. In addition, the identification of electrogram amplitude gradients has important clinical ramifications for unipolar electrogram studies such as noncontact or dynamic substrate mapping. For example, unipolar voltage mapping, using V_P-P_ was less dependent on distance from the pacing stimulus than unipolar V_-P_, and hence might provide superior accuracy in more efficaciously localising ventricular scar. Moreover, our results suggest that the number of pacing sites should be increased and spaced closer together to minimise distance related effects for use of V_-P_, such as that employed by dynamic substrate mapping of scar [1], [31]. Specifically, appropriate selection of recording modalities should be considered during LV mapping in situations associated with non-ischemic dilated cardiomyopathy attributing to low amplitude substrate at the base of the LV, or in patients with left bundle branch block. In both situations, unipolar mapping during sinus rhythm would be most affected due to increased wavefront propagation distance. Low LV ejection fraction due to LV dilatation is often a hallmark feature of non-ischemic cardiomyopathy. The hypothesis relating to propagation distance is supported by clinical studies, indicating that when comparing endocardial voltage substrate maps between patients with low LV ejection fraction (35±14%) and non-ischemic cardiomyopathy, to patients with near normal LV ejection fraction (48±13%) and non-ischemic cardiomyopathy, whilst there were no differences in bipolar amplitude or LV wall thickness between groups, a significant reduction in unipolar amplitude was observed in the low LV ejection fraction group [32]. Finally, it is likely that higher amplitudes may be reported during unipolar mapping of the epicardium.

### Limitations

Plunge needles used for electrogram recordings in this study are not commonly used for clinical mapping. However, the intramural needles enabled accurate 3-dimensional evaluation of the effects of recording distance and pacing depth. The plunge needles used in this study were not fixed to each other, and hence were not inhibitory to the mechanical motion of myocardium. Previous *In Vivo* studies have shown that unipolar electrogram morphology, timing and gross histological assessment of structure and risk of inflammation were not significantly affected by short and long term insertion of multiple transmural electrodes, in Canine ventricles [8], [15], [33].

Experiments were performed in an open chest model that may predispose to distortion of epicardial conductivity; however the heart surface was maintained moist throughout the procedure. The purkinje network in sheep have been reported to be more extensively distributed at the sub-endocardium than in humans [34]. However, we specifically analysed ventricular paced beats that did not have competing purkinje derived stimulation during atrial activation.

Finally none of the animals had scar. Whether the reported results can be generalized to ventricles with scar (ischemic or non-ischemic) warrants further investigation.

## Supporting Information

Checklist S1 **ARRIVE Checklist.**
(DOC)
